# Advanced Practice Nurses' Evidence‐Based Healthcare Competence and Associated Factors in Finland and Singapore—A Cross‐Sectional Study

**DOI:** 10.1111/jan.16771

**Published:** 2025-01-24

**Authors:** Saija Ylimäki, Anne Oikarinen, Anna‐Maria Tuomikoski, Brigitte Woo, Kristina Mikkonen, Wentao Zhou, Heidi Parisod, Sami Sneck, Hannu Vähänikkilä, Maria Kääriäinen

**Affiliations:** ^1^ Research Unit of Health Sciences and Technology Oulu Finland; ^2^ Oulaskangas Hospital The Wellbeing Services County of North Ostrobothnia Oulu Finland; ^3^ Oulu University Hospital The Wellbeing Services County of North Ostrobothnia Oulu Finland; ^4^ Nursing Research Foundation Helsinki Finland; ^5^ Medical Research Center Oulu Oulu Finland; ^6^ Alice Lee Centre for Nursing Studies National University of Singapore (NUS) Singapore Singapore; ^7^ The Finnish Centre for Evidence‐Based Health Care: A JBI Centre of Excellence Helsinki Finland; ^8^ University of Turku Department of Nursing Science Turku Finland; ^9^ Northern Finland Birth Cohorts, Faculty of Medicine University of Oulu Oulu Finland

**Keywords:** advanced practice nurse, competence, cross‐sectional study, evidence‐based healthcare, nursing

## Abstract

**Aim:**

To describe and compare the Evidence‐Based HealthCare (EBHC) competence of Advanced Practice Nurses (APNs), and the factors associated with it in Finland and Singapore.

**Design:**

A descriptive and analytical cross‐sectional study.

**Methods:**

Data were collected from APNs working in healthcare in Finland (*n* = 157) or Singapore (*n* = 99) between May 2023 and October 2023 using a self‐assessment instrument to measure EBHC competence (EBHC‐Comp‐APN) and an EBHC knowledge test. The data were analysed using descriptive statistics, analysis of variance, K‐mean cluster and multivariate analyses.

**Results:**

The self‐assessments of APNs working in Finland and Singapore regarding their EBHC competence level varied and three distinct profiles of APNs' EBHC competence were identified in both countries. The strongest EBHC competence was in ‘The Knowledge Needs Related to Global Health’, while the weakest in ‘Evidence Synthesis and Transfer’. The country‐specific differences were identified in factors associated with EBHC competence.

**Conclusion:**

The EBHC competencies of APNs vary widely and require planned and needs‐driven development. In connection with the development of EBHC competence, the factors related to competence should be considered country‐by‐country.

**Implications for the Profession:**

The APN's EBHC competence should be systematically developed considering the factors associated with and the current level of EBHC competence.

**Impact:**

The level of EBHC competence of APNs and associated factors should be identified when developing their competence and role in collaboration with APNs, leaders of healthcare and education organisations and policy makers. In addition, research into APNs' EBHC competence should continue.

**Reporting Method:**

The STROBE checklist was used in the reporting of the study.

**Patient or Public Contribution:**

No patient or public contribution.

**Summary**

- What does this paper contribute to the wider global clinical community?
  ○ The study highlights that the primary development targets for the EBHC competence of APNs working in Finland and Singapore are the competence in evidence synthesis and transfer.
  ○ The study indicated differences in EBHC competence and associated factors among APNs working in Finland and Singapore. Thus, the development of the role of APNs should occur at the national level, but it necessitates international cooperation and research.
  ○ The study identified that APNs' participation in research projects and national networks of experts and working in the nursing guideline group and leadership tasks (projects or staff) strengthened the EBHC competence of Finnish APNs.

## 1 Introduction

Evidence‐based implementation of healthcare in a rapidly changing and developing environment ensures high‐quality, safe, cost‐effective (Aiken et al. 2014; Melnyk et al. 2014) and feasible, appropriate, meaningful and effective healthcare services (Jordan et al. 2019). Advanced practice nurses (APNs) play a significant and extensive role in promoting evidence‐based healthcare (EBHC) in clinical environments (Schober et al. 2020). APNs' EBHC tasks include critical appraisal, evidence transfer and implementation, monitoring the use of evidence, practice harmonisation, development evidence‐based practice (EBP) and providing support e.g., through education. Additionally, APNs' responsibilities involve carrying out independent patient care; supporting the organisation's staff in implementing, promoting and enabling evidence‐based healthcare, and leading multidisciplinary development teams or projects both within the organisation and nationally. They are also responsible for transferring evidence to political decision‐makers at national and international levels (Jokiniemi et al. 2020; Schober et al. 2020; Ylimäki et al. 2022). APNs need extensive and deep competence in every sub‐dimension of EBHC to promote its implementation in decision‐making about the guidance, rehabilitation and treatment of patients/clients (Ylimäki et al. 2024).

## 2 Background

The Joanna Briggs Institute (JBI) Model of evidence‐based healthcare identifies five critical areas essential for achieving EBHC: global health, generation, synthesis, transfer and implementation of evidence (Jordan et al. 2019). Global health requires that APNs are competent in identifying information needs, critically evaluating current practices, engaging with EBHC and maintaining a positive attitude towards it (Jordan et al. 2019; Ylimäki et al. 2022). Evidence generation requires competence in using information technology (Bhatarasakoon et al. 2022), generating research data, such as information about their organisation's systems or other contexts (Melnyk et al. 2014; Schober et al. 2020; Woo et al. 2019) and understanding different sources of evidence such as discourse, expertise and research (Jordan et al. 2019). For evidence synthesis, APNs must be competent in searching for evidence (Ylimäki et al. 2022), formulating research questions, evaluating the quality of existing studies (Bhatarasakoon et al. 2022; Gerrish et al. 2011), conducting systematic reviews and producing evidence summaries and guidelines (Jordan et al. 2019; Malik, McKenna, and Plummer 2015; Schober et al. 2020; Ylimäki et al. 2022).

To support evidence transfer, APNs need expertise in publishing research (Woo et al. 2019), implementing education (Jordan et al. 2019; Schober et al. 2020; Woo et al. 2019), promoting the integration of evidence into patient information systems (Jordan et al. 2019; Schober et al. 2020) and actively disseminating evidence (Jordan et al. 2019; Malik, McKenna, and Plummer 2015; Schober et al. 2020). Competence in evidence implementation involves using evidence in decision‐making related to patient care, rehabilitation or guidance (Bhatarasakoon et al. 2022; Malik, McKenna, and Plummer 2015; Schober et al. 2020; Woo et al. 2019). It also includes developing evidence‐based practices, conducting context analysis (Jordan et al. 2019; Ylimäki et al. 2022), facilitating change (Gerrish et al. 2011; Jordan et al. 2019; Ylimäki et al. 2022; Woo et al. 2019) and evaluating the process and outcomes of change (Jordan et al. 2019; Ylimäki et al. 2022; Ylimäki et al. 2024). A systematic review by Ylimäki et al. (2024) found that higher level of education (at least master's degree) in APNs is associated with improved EBHC competence. In contrast, factors such as marital status, gender, nationality, work experience, job title, weekly working hours, academic environment, Magnet hospital status and research council involvement showed limited or contradictory relationships with EBHC competence (Ylimäki et al. 2024).

According to ICN, APN is an umbrella term for nurses who have acquired an advanced knowledge base, complex decision‐making skills and clinical competence through additional education (at least a master's degree), with roles and competence shaped by the specific context in which they are credentialed to practice (Schober et al. 2020). In practice, however, the educational background, job descriptions and job titles of APNs vary due to differences in regulations and the phasing of introducing APN job descriptions in different countries (Dowling et al. 2013; Janson, Opheim, and Hellesø 2023; Jean and Briana 2019). In Finland, tasks with extended responsibilities and advanced nursing are performed by CNS, NP (Jokiniemi et al. 2022) and part of registered nurses and special registered nurses (De Raeve et al. 2024). Similarly, in Singapore, not only APNs (Xu et al. 2022), but also nurse managers, nurse educators and nurse clinicians carry out extended and advanced nursing (Ministry of Health (MOH) Singapore 2024). This study's target population included those performing extended and advanced nursing, and they are referred to as an APN.

The development of extended and advanced tasks in nursing began in both countries at the beginning of the 2000s (De Raeve et al. 2024; Jokiniemi et al. 2022; Kannusamy 2006; Xu et al. 2022). In Finland, APN education is organised in several universities of applied sciences and universities (Jokiniemi et al. 2020), in Singapore the education is concentrated in one university (Ayre and Bee 2014) and two universities of applied sciences (Care to go Beyond 2024). APNs working in Singapore were selected in this study because the study aimed to provide new insights into the evidence‐based nursing competence of Finnish APNs for development and management. Singapore has the best healthcare system in the world (International Citizens Insurance 2024), and the health and education of its population are also rated as the best (Legatum Institute 2024). Finland was ranked second best in population education and the 15th best country in population health of Legatum prosperity Index (Legatum Institute 2024). We suggest that it is worth investigating whether these differences have any association with APNs level of EBHC competence, particularly considering an earlier finding that the EBHC competence of APNs has not been studied adequately using the JBI EBHC model and the relevant job descriptions (Ylimäki et al. 2024). This study aimed to fill this gap.

## 3 The Study

The purpose of the study was to describe and compare Advanced Practice Nurses' (APNs) Evidence‐Based HealthCare (EBHC) competence profiles, and the factors associated with them in Finland and Singapore. The research questions were as follows:

1. What are the levels and profiles of EBHC competencies among APNs in Finland and Singapore?
2. What factors are associated with EBHC competence and profiles of EBHC competence among APNs in Finland and Singapore?
3. What are the differences in APNs EBHC competence between Finland and Singapore?

## 4 Methods

### 4.1 Study Design

A descriptive and analytical cross‐sectional study was conducted between two countries, Finland and Singapore (Cory 2019; Kesmodel 2018; Maier et al. 2023; Polit and Beck 2017). The study followed the STROBE checklist which guided the study's reporting (von Elm et al. 2007; Data S1).

### 4.2 Study Participants

All registered and senior nurses who had an advanced job description related to the APN role were invited to the study. In Finland, all CNSs, and registered nurses with advanced job descriptions in clinical practice at five university hospitals and one primary healthcare service were invited to participate in the study. In Singapore, all APNs, and senior nurses with advanced job descriptions such as assistant nurse clinicians (ANC), nurse clinicians (NC), senior nurse clinicians (SNC), nurse managers (NM), senior nurse managers (SNM), nurse educators (NE) and senior nurse educators (SNE) in clinical practice were invited to participate. The inclusion criteria for the study were (1) APN or a registered/senior nurse with an advanced job description in clinical practice, (2) working in primary or special healthcare and (3) working full‐time or part‐time. The inclusion criteria were determined in this way because the available registry data suggested that the number of registered APNs was limited in both countries, and we wanted to get information about EBHC competence from all nurses performing an advanced job description. Registry data was only available for CNSs (*N* = 120) in Finland and APNs (*N* = 383) in Singapore; no registry data were available for other job titles (Finnish Nurses Association (FNA) 2023; Ministry of Health (MOH) Singapore 2023).

### 4.3 Instruments

Data were collected using a new EBHC‐Comp‐APN instrument and an existing EBHC knowledge test. The instrument was developed because existing tools for measuring evidence‐based practice did not account for the full scope of tasks performed by APNs, nor did they cover all the key areas of the EBHC model. The newly developed self‐assessment instrument (EBHC‐Comp‐APN) measures the EBHC competence of APNs. The instrument was developed at the University of Oulu and the Nursing Research Foundation. The development of the instrument will be published elsewhere. It is based on the JBI model of EBHC (Jordan et al. 2019) and considers the results of systematic reviews which relate to the general EBHC competence of nurses (Härkönen et al. 2021), advanced practice nurses (Ylimäki et al. 2024), educators (Immonen et al. 2022) and leaders (Koivunen et al. 2023). In addition, the results of a qualitative study (Ylimäki et al. 2022) and the ICN guidelines on advanced practice nursing (Schober et al. 2020) were considered in the context of the formation of the items describing the EBHC competence concerning ‘the role of APNs in EBHC’. The content validity of the instrument was evaluated by EBHC experts (*n* = 9) (master's level nursing educators, nursing leaders, researchers, CNSs). Overall, the instrument showed excellent content validity S‐CVE = 0.99 (Kääriäinen, Mikkonen, and Kyngäs 2020). The instrument was piloted with graduating Master of Nursing Science students (*n* = 22) who rated its functionality and clarity. In addition, the average response time was calculated. The construct validity of the instrument was examined by explorative factor analysis which supported a five‐factor model. Detailed development and testing of the instrument will be reported elsewhere.

The questionnaire contained 16 background questions, 61 items from EBHC‐Comp‐APN and 11 items from the EBHC knowledge test. The EBHC‐Comp‐APN instrument contained 5 sub‐dimensions: ‘the knowledge needs related to global health’ (10 items); ‘evidence generation’ (14 items); ‘evidence synthesis and transfer’ (8 items); and ‘evidence implementation’ (13 items) and the new sub‐dimension ‘the role of APNs in EBHC’ (16 items). The items were measured on a five‐point Likert rating scale (1‐ very poor, 2‐ poor, 3‐ fair, 4‐ good and 5‐ excellent competence). Background questions concerned APNs' age, gender, previous education, year of completion of the highest degree, current job title, place of current practice, continuing education level, additional qualifications, participation in research projects and participation in development projects, and organisation‐specific background. The country, in which the participant's worked (Finland or Singapore) was also considered as a background factor. Cronbach's alpha values for the EBHC‐Comp‐APN instruments (English and Finnish) ranged from 0.98 to 0.99, and Cronbach's alpha values for the sub‐dimensions ranged from 0.91 to 0.97. The instrument showed excellent internal consistency (De Von et al. 2007; Kääriäinen, Mikkonen, and Kyngäs 2020; Polit and Beck 2017).

The EBHC knowledge test included eleven questions with the response options ‘Yes’, ‘No’ or ‘I don't know’. The EBHC knowledge test was developed collaboratively by the University of Oulu and the Nursing Research Foundation and has previously been used in national‐level EBHC competence surveys in Finland. Responses were scored according to the percentage of questions correctly answered.

The EBHC‐Comp‐APN instrument, questionnaire and EBHC knowledge test were originally in Finnish. These were translated into English in three stages by four researchers: (1) the instrument, questionnaire and test were translated from Finnish to English by two independent researchers, and translation differences were resolved by consensus among three researchers; (2) the fourth researcher translated the instrument, questionnaire and test from English to Finnish and (3) the re‐translated Finnish version was compared for consistency with the original version. The translations were accepted because they were mutually consistent. In addition, a fifth researcher with excellent English checked the instrument, questionnaire and test. Finally, together with Singaporean researchers, word‐level revisions and specifications were made to the English versions to make the EBHC‐Comp‐APN instrument, the questionnaire and the EBHC knowledge test statements understandable to APNs working in Singapore.

### 4.4 Data Collection

The data were collected through Webropol and Qualtrics surveys in May–October 2023. In Finland, the survey was sent to APNs by e‐mail via contact persons in the relevant organisations. In Singapore, the survey was sent via the NUS Nursing Alumni email database. In the e‐mail, participants received a data protection form and information about the eligibility conditions, the purpose of the study and the main content of the survey. Only participants who gave informed consent were able to answer the survey. Participants were given 5 weeks to answer the survey, during which the participating organisations were advised to issue 3 reminders. The response rate could not be calculated because there was no statistical information on the size of the target population in Finland and Singapore.

### 4.5 Data Analysis

Data were analysed using IBM SPSS Statistics 29.0 (IBM Corp., 2022). Descriptive statistics frequency (f), percentage (%), mean, median and standard deviation (SD) values were used to describe participants' characteristics and their level of EBHC competence. The K‐mean cluster algorithm was used to identify EBHC profiles. Cluster analysis was used to identify sub‐groups of the data used whose members are similar in terms of EBHC competence characteristics while at the same time differing from members of other groups. The EBHC competence levels of these profiles were interpreted on a five‐point Likert scale, where a mean of ≤ 2.49 indicates low competence, 2.50–3.49 is moderate, 3.50–4.49 is good, and a mean of ≥ 4.50 is excellent. The EBHC knowledge test results of the profiles have been interpreted according to the scores of the answers: ≤ 69.9% means poor EBHC knowledge, 70%–79.9% moderate, 80%–89.9% good and ≥ 90% excellent. Dependence between classified background variables, overall competence and profiles was analysed with crosstabs, and Chi‐square test. Dependence between continuous background variables, overall competence and clusters was analysed with a one‐way analysis of variables (ANOVA), independent samples *t*‐test, Mann–Whitney's *U* and Kruskal–Wallis tests. When a statistically significant difference between the investigated groups was identified, a Bonferroni correction was applied to evaluate whether each group differed significantly.

Multivariate logistic regression was used to determine the independent effect of variables affecting APNs' EBHC competence by adjusting the effect of other variables affecting EBHC competence. For the multivariate logistic regression analysis, the data were transferred to dichotomous form (0 = 1–3.49—learners; 1 = 3.5–5—experts) to enable analysis. Four multivariate logistic regression analyses were constructed for (1) personal factors, (2) participation‐related factors, (3) working‐related factors and (4) organisational factors. In the multivariate logistic regression analysis constructed for sociodemographic factors, the year of graduation from the highest degree and job title were excluded due to multicollinearity (Polit and Beck 2017).

The statistical significance level was set at *p* < 0.05 in all the tests conducted (Polit and Beck 2017). 0.6% of all answers had missing values but these were not replaced in the analyses. No analysis of missing values was performed because the number of missing responses was very small. Variation in the number of participants was allowed in the analyses, and the numbers of participants were reported.

### 4.6 Ethical Considerations

The research generally followed the principles of good scientific research, which include applying for a research permit, accurately describing the entire research process, appropriate citation of references, stating important conflicts of interest, and clarifying the rights and obligations of the research parties (World Medical Association 2013). Research permits were applied for and approved in Finland and Singapore in according with local practice. A research permission for the study was obtained from the National University of Singapore's Institutional Review Board (NUS‐IRB‐2023‐645). According to Finnish ethical research standards, no statement from the Research Ethics Committee was required because the study did not concern the physical or psychological integrity of health professionals (TENK 2023). All participants in the study gave their informed consent before completing the survey. The research followed the European Union's General Data Protection Regulation (General Data Protection Regulation (GDPR) 2016). All participants' personal information was kept confidential, and their anonymity in reporting was ensured.

## 5 Results

### 5.1 Participants' Characteristics

Participants in the study were APNs or similar registered or senior nurses with advanced from Finland (*n* = 157) and Singapore (*n* = 99) with an advanced clinical role in clinical practice. Table 1 provides detailed information on the characteristics of the participants. There were statistically significant differences between APNs working in Finland and Singapore regarding age, education level, year of completion of the highest degree, job titles, current field of work, as well as participation in research and development projects (Table 1).

**TABLE 1:** Participants' demographic characteristics in Finland and Singapore.

Characteristics	Finland (*n* = 157)	Singapore (*n* = 99)	*p*
Age in years, mean ± SD	46.78 ± 9.78	36.00 ± 11.00^d^	**0.001** ^**c**^
Gender, *n* (%)			0.051^b^
Female	149 (94.9)	87 (87.9)	
Male	6 (3.8)	11 (11.1)	
Do not want to tell	2 (1.3)	1 (1.0)	
Education level, *n* (%)			**0.004** ^**b**^
Vocational degree (Applied Sciences and University Bachelor's degree)	112 (71.3)	51 (51.5)	
Master's degree (Applied Sciences and University Master's degree)	41 (26.1)	45 (45.5)	
Doctoral degree (University degree)	4 (2.5)	3 (3.0)	
The year of completion of the highest degree, mean ± SD	2008.72 ± 10.72	2015.00 ± 9.00^d^	**< 0.001** ^**c**^
Job title, *n* (%)			**< 0.001** ^**b**^
Registered Nurses and Specialist Nurses	79 (50.3)	0 (0.0)	
Nurse Clinicians	0 (0.0)	52 (52.5)	
Nurse Practitioners	47 (29.9)	0 (0.0)	
Nurse managers and Nurse educators	0 (0.0)	26 (26.3)	
Clinical Nurse Specialists	30 (19.1)	0 (0.0)	
Advanced Practice Nurses	1 (0.6)	21 (21.2)	
Current APNs work field, *n* (%)			**< 0.001** ^**a**^
Specialised social‐ and healthcare (Acute care in hospital, Outpatient in hospital)	86 (54.8)	84 (84.8)	
Primary social‐ and healthcare (Community hospital, Polyclinic and Home Care)	84 (45.2)	15 (15.2)	
Participated in development and research projects, *n* (%)			
Participated in development projects	106 (67.5)	86 (86.9)	**< 0.001** ^**a**^
Participated in research projects	33 (21.0)	67 (67.7)	**< 0.001** ^**a**^

*Note:* Bold values indicate *P* < 0.05.

Abbreviation: SD, standard deviation.

^a^ Pearson Chi‐Square Test.

^b^ Fisher Chi‐Square Test.

^c^ Mann–Whitney *U* test.

^d^ Median (IQR = Interquartile Range).

### 5.2 Participants' EBHC Competence Level and Profiles in Finland

The EBHC competence level of APNs working in Finland (*n* = 157) varied across five sub‐dimensions: ‘the knowledge needs related to global health’ (mean 3.49, SD 0.72), ‘evidence generation’ (mean 3.03, SD 0.77), ‘evidence synthesis and transfer’ (mean 2.38, SD 0.93), ‘evidence implementation’ (mean 3.09, SD 0.94) and ‘the role of APNs in EBHC’ (mean 2.68, SD 0.94). The median score on the EBHC knowledge test was 75% (median) but individual results varied considerably (IQR = 25.00, see Table 2).

**TABLE 2:** Participants' EBHC competence in Finland and Singapore.

EBHC competence	Finland (*n* = 157)	Singapore (*n* = 99)	*p*
The knowledge needs related to global health, mean ± SD	3.49 ± 0.72	3.44 ± 0.58	0.487^a^
Evidence generation, mean ± SD	3.03 ± 0.77	3.13 ± 0.49	0.291^a^
Evidence synthesis and transfer, mean ± SD	2.38 ± 0.93	3.07 ± 0.65	**< 0.001** ^**a**^
Evidence implementation, mean ± SD	3.09 ± 0.94	3.26 ± 0.58^c^	**0.034** ^**a**^
The role of APNs in EBHC, mean ± SD	2.68 ± 0.94	3.23 ± 0.63^c^	**< 0.001** ^**a**^
APNs overall EHBC competence, scale 1–5, mean ± SD	**2.92 ± 0.78**	**3.23 ± 0.52** ^**c**^	**< 0.001** ^**a**^
Knowledge test about EBHC, median ± IQR	75.00 ± 25.00	82.00 ± 9.000^d^	0.599^b^

*Note:* Bold values indicate *P* < 0.05.

Abbreviations: IQR, interquartile range; SD, standard deviation.

^a^ Independent Samples Test.

^b^ Mann–Whitney *U* test.

^c^ *n* = 93.

^d^ *n* = 90.

Participants' EBHC competence was presented in three different profiles ranging from an average score of 2.01 to 3.80. A three‐profile model was found to be most appropriate to represent APNs' EBHC competence levels. EBHC competence differed between profiles to a statistically significant degree (*p* < 0.001) on all five sub‐dimensions (Table 3). In all profiles, APNs evaluated their competence in ‘the knowledge needs related to global health’ as the highest and in ‘evidence synthesis and transfer’ as the lowest. Overall, EBHC competence was highest in Profile A, moderate in Profile B and lowest in Profile C.

**TABLE 3:** Participants' EBHC competence profiles in Finland.

Characteristics and competence	Profile A (*n* = 50)	Profile B (*n* = 58)	Profile C (*n* = 49)	*p*
Age in years, mean ± SD	45.0 ± 8.9	46.2 ± 9.7	49.3 ± 10.4	0.080^c^
Gender, *n* (%)				0.941^a^
Female	47 (94.0)	56 (96.6)	46 (93.9)	
Male	2 (3.4)	2 (3.4)	2 (4.1)	
Dont want to tell	1 (2.0)	0 (0.0)	1 (2.0)	
Education level, *n* (%)				**< 0.001** ^**b**^
Vocational degree (Applied Sciences and University Bachelor's degree)	17 (34.0)	47 (81.0)	48 (98.0)	
Master's degree (Applied Sciences and University Master's degree)	29 (58.0)	11 (19.0)	1 (2.0)	
Doctoral degree (University degree)	4 (8.0)	0 (0.0)	0 (0.0)	
The year of completion of the highest degree (mean ± SD, ″=median ± IQR)	2017.0 ± 9.0”	2006.9 ± 10.7	2004.9 ± 10.6	**< 0.001** ^**c**^
Job title, *n* (%)				**< 0.001** ^**a**^
Registered Nurses and Specialist Nurses	17 (34.0)	30 (51.7)	32 (65.3)	
Nurse Clinicians	0 (0.0)	0 (0.0)	0 (0.0)	
Nurse Practitioners	12 (32.8)	19 (32.8)	16 (32.7)	
Nurse managers and Nurse educators	0 (0.0)	0 (0.0)	0 (0.0)	
Clinical Nurse Specialists	20 (40.0)	9 (15.5)	1 (2.0)	
Advanced Practice Nurses	1 (2.0)	0 (0.0)	0 (0.0)	
Current APNs work field, *n* (%)				**0.009** ^**b**^
Specialised social‐ and healthcare (Acute care in hospital, Outpatient in hospital)	32 (64.0)	36 (62.1)	18 (36.7)	
Primary social‐ and healthcare (Community hospital, Polyclinic and Home Care)	18 (36.0)	22 (37.9)	31 (63.3)	
Participated in development and research projects, *n* (%)				
Participated in development projects	40 (80.0)	44 (75.9)	22 (44.9)	**< 0.001** ^**b**^
Participated in research projects	20 (40.0)	9 (15.5)	4 (8.2)	**< 0.001** ^**b**^
EBHC competences, mean ± SD				
The Knowledge Needs Related to Global Health	4.01 ± 0.42	3.54 ± 0.49	2.78 ± 0.53	**< 0.001** ^**c**^
Evidence Generation	3.80 ± 0.43	3.08 ± 0.42	2.19 ± 0.41	**< 0.001** ^**c**^
Evidence Synthesis and Transfer	3.35 ± 0.71	2.33 ± 0.48	1.45 ± 0.41	**< 0.001** ^**c**^
Evidence Implementation	3.99 ± 0.41	3.17 ± 0.49	1.95 ± 0.49	**< 0.001** ^**c**^
The role of APNs in EBHC	3.73 ± 0.49	2.62 ± 0.44	1.68 ± 0.42	**< 0.001** ^**c**^
APNs overall EHBC competence, scale 1–5, mean ± SD	**3.80 ± 0.35**	**2.94 ± 0.25**	**2.01 ± 0.32**	**< 0.001** ^**c**^
Knowledge test about EBHC, mean ± SD	83.0 ± 9.0”	75.0 ± 16.0”	62.5 ± 22.5	**< 0.001** ^**c**^

*Note:* Bold values indicate *P* < 0.05.

^a^ Fisher–Freeman–Halton Exact test.

^b^ Pearson Chi‐Square test.

^c^ One‐way ANOVA.

Profile A included 31.8% (*n* = 50) of all participants and displayed the highest averages on the EBHC knowledge test and EBHC competence (mean variation of sub‐dimensions 3.35–4.01). EBHC competence was good at ≥ 3.5 in all other sub‐dimensions apart from ‘evidence synthesis and transfer’. The mean age of APNs was 45.0 years, and the median year of graduation from their highest degree was 2017. Profile A had the most doctoral (8.0%) and master's degrees (58.0%). Most APNs in this profile worked in specialised healthcare (64.0%). Profile A also included the most clinical nurse specialists (40.0%). Most of them had participated in development projects (80.0%) and research projects (40.0%) (Table 3). Over the previous two years, 48.0% of the Profile A APNs had participated in a national expert network and 30.0% in scientific conferences; further, 20.0% of the APNs in this profile had worked in a nursing guideline group, 36.0% as a producer or assistant of electronic education material, and 32.0% as a leader of projects or a department (Table S1).

Profile B represented the largest number of participants at 36.9% (*n* = 58). APNs in this profile rate their EBHC competence as moderate, both overall (mean 2.94) and in each sub‐dimension (mean variation 2.62–3.54) except for Evidence Synthesis and Transfer (2.33) sub‐dimension. The mean age of this profile was 46.2 years, and the mean year of completion of the highest degree was 2007 (Table 3) Profile B APNs participated most often networks within your own organisation (60.3%), their organisations most often had a research council (41.4%), teamwork supported the development of EBP (82.8%), and clear tasks and roles for different nursing professionals, e.g., nurse, nurse practitioner and APN (27.6%). Profile B had the least number of people who participated in continuing education offered by an external organisation (53.4%) (Table S1).

Profile C included 31.2% (*n* = 49) of the APNs. Profile C evaluated their overall EBHC competence as low at 2.01 (mean), with mean variation in sub‐dimensions 1.45–2.78. Only the ‘the knowledge needs related to global health’ competence was assessed as moderate (mean 2.78). The result of the EBHC knowledge test was also poor. The average age of this profile was 49.3 years and the average year of completion of the highest degree was 2005. Most of the Profile C participants had a vocational degree (98.0%) and worked in primary healthcare (63.3%) (Table 3). Profile C APNs had participated the least in development and research projects, scientific conferences and the national expert network. In Profile C, most participants participated in continuing education that was offered by your own organisation (59.2%), and most felt that the roles of different experts and management in the developing evidence‐based practice were clearly defined (83.7%) (Table S1).

The level of EBHC competence had a statistically significant positive association with higher education level, most recent year of highest degree completion, job title CNS (versus registered nurse, specialist nurse), specialised care setting (vs. primary healthcare settings) and more active participation in research and development projects, scientific conferences, your own organisation's networks, national networks and following professional social media. In addition, the level of EBHC competence was positively associated with working in a nursing guideline group, producing written and electronic learning material, acting as an educator, mentor or consultant, and managing projects or a department. Further, several background factors relating to the organisation had a positive and statistically significant association with APNs' EBHC competence profile or level; (1) EBHC is documented in the strategy, (2) management supports EBHC, (3) teamwork supports EBHC, (4) sufficient resources to develop evidence‐based standard operating practice, (5) opportunity to participate in the development of EBHC, (6) journal club activities, (7) research council activities, (8) existing data (e.g., statistics, registers) systematic uses for the development of services (Table 3; Table S1).

### 5.3 Participants' EBHC Competence Level and Profiles in Singapore

The EBHC competence level of APNs working in Singapore (*n* = 99) varied across five sub‐dimensions: ‘the knowledge needs related to global health’ (mean 3.44, SD 0.58), ‘evidence generation’ (mean 3.13, SD 0.49), ‘evidence synthesis and transfer’ (mean 3.07, SD 0.65), ‘evidence implementation’ (mean 3.26, SD 0.58) and ‘the role of APNs in EBHC’ (mean 3.23, SD 0.63). The median score on the EBHC knowledge test was 82% (median) but individual results varied considerably (IQR = 9.00, see Table 2).

Participants' EBHC competence was presented in three different profiles ranging from an average score of 2.55 to 3.81. A three‐profile model was identified as the most suitable for representing the EBHC competence levels of APNs. EBHC competence differed between profiles to a statistically significant degree (*p* < 0.001) on all five sub‐dimensions (Table 4). In all profiles, APNs evaluated their competence in ‘the knowledge needs related to global health’ as the highest. In Profiles A and B, ‘synthesis and transfer of evidence’ was evaluated as the lowest competence, and in Profile C, ‘the role of APNs in EBHC’. EBHC competence was highest in Profile A, moderate in Profile B and lowest in Profile C.

**TABLE 4:** Participants' EBHC competence profiles in Singapore.

Characteristics and competence	Profile A (*n* = 27)	Profile B (*n* = 44)	Profile C (*n* = 22)	*p*
Age in years, mean ± SD	39.8 ± 10.2	35.5 ± 5.0^d^	40.5 ± 12.0^d^	0.285^c^
Gender, *n* (%)				**0.026** ^**a**^
Female	20 (74.1)	40 (90.9)	22 (100)	
Male	6 (22.2)	4 (9.1)	0 (0.0)	
Dont want to tell	1 (3.7)	0 (0.0)	0 (0.0)	
Education level, *n* (%)				0.093^a^
Vocational degree (Applied Sciences and University Bachelor's degree)	14 (51.9)	17 (38.6)	15 (68.2)	
Master's degree (Applied Sciences and University Master's degree)	11 (38.6)	26 (59.1)	7 (31.8)	
Doctoral degree (University degree)	2 (7.4)	1 (2.3)	0 (0.0)	
The year of completion of the highest degree (mean ± SD, “=median ± IQR)	2017.0 ± 9.0	2010.8 ± 9.8	2012.0 ± 8.0”	**0.009** ^**c**^
Job title, *n* (%)				0.085^a^
Registered Nurses and Specialist Nurses	0 (0.0)	0 (0.0)	0 (0.0)	
Nurse Clinicians	13 (48.1)	19 (43.2)	16 (72.7)	
Nurse Practitioners	0 (0.0)	0 (0.0)	0 (0.0)	
Nurse managers and Nurse educators	10 (37.0)	12 (27.3)	2 (9.1)	
Clinical Nurse Specialists	0 (0.0)	0 (0.0)	0 (0.0)	
Advanced Practice Nurses	4 (14.8)	13 (29.5)	4 (18.2)	
Current APNs work field, *n* (%)				0.926^a^
Specialised social‐ and healthcare (Acute care in hospital, Outpatient in hospital)	24 (88.9)	37 (84.1)	19 (86.4)	
Primary social‐ and healthcare (Community hospital, Polyclinic and Home Care)	3 (11.1)	7 (15.9)	3 (13.6)	
Participated in development and research projects, *n* (%)				
Participated in development projects	24 (88.9)	39 (88.6)	18 (81.8)	0.722^a^
Participated in research projects	19 (70.4)	31 (70.5)	14 (63.6)	0.879^b^
EBHC competences, mean ± SD				
The Knowledge Needs Related to Global Health	4.00 ± 0.45	3.40 ± 0.38	2.84 ± 0.36	**< 0.001** ^**c**^
Evidence Generation	3.63 ± 0.33	3.06 ± 0.32	2.62 ± 0.30	**< 0.001** ^**c**^
Evidence Synthesis and Transfer	3.64 ± 0.52	3.06 ± 0.42	2.57 ± 0.57”	**< 0.001** ^**c**^
Evidence Implementation	3.88 ± 0.42”	3.29 ± 0.25	2.67 ± 0.42”	**< 0.001** ^**c**^
The role of APNs in EBHC	3.94 ± 0.19”	3.18 ± 0.28	2.50 ± 0.34”	**< 0.001** ^**c**^
APNs overall EHBC competence, scale 1–5, mean ± SD	**3.81 ± 0.39”**	**3.20 ± 0.21**	**2.55 ± 0.20**	**< 0.001** ^**c**^
Knowledge test about EBHC, mean ± SD	82.69 ± 8.02	82.00 ± 9.00”	73.00 ± 18.00”	**0.032** ^**c**^

*Note:* Bold values indicate *P* < 0.05.

^a^ Fisher–Freeman–Halton Exact test.

^b^ Pearson Chi‐Square test.

^c^ One‐way anova.

^d^ =median ± IQR.

Profile A included 29% (*n* = 27) of all participants and displayed the highest averages on the EBHC knowledge test and EBHC competence (mean variation of sub‐dimensions 3.63–4.00). EBHC competence was good at ≥ 3.5 in all sub‐dimensions. The mean age of APNs was 39.8 years, and the median year of graduation from their highest degree was 2017. Profile A had the most doctoral (7.4%). Most APNs in this profile worked in specialised healthcare (88.9%). Profile A also included the most nurse managers and educators (37.0%). Most of them had participated in development projects (88.9%) (Table 4). 44.4% of the APNs in this profile had worked as a mentor within the organisation, and 59.3% as a leader of projects or a department (Table S2).

Profile B represented the largest number of participants at 47.3% (*n* = 44). APNs in this profile rate their EBHC competence as moderate, both overall (mean 2.94) and in each sub‐dimension (mean variation 3.06–3.40). The mean age of this profile was 35.5 years, and the mean year of completion of the highest degree was 2011. Profile B included the most APNs (29.5%) (Table 4).

Profile C included 23.7% (*n* = 22) of the APNs. Profile C evaluated their overall EBHC competence as moderate at 2.55 (mean), with mean variation in sub‐dimensions 2.50–2.84. The EBHC knowledge test result was also moderate. The average age of this profile was 40.5 years and the average year of completion of the highest degree was 2012. Most of the Profile C participants had only a vocational degree (68.2%) (Table 4). Profile C APNs had participated the least mentoring in their organisation and in leadership (in projects or the department). In Profile C, most participants participated in continuing education that was offered by an external organisation (45.5%), and most felt that the roles of different experts and management in the developing evidence‐based practice were not clearly defined (72.7%) (Table S2).

Among the personal background factors, gender and year of highest degree completion was statistically significantly associated with the EBHC competency profile of APNs working in Singapore. In addition, the level of EBHC competence was positively associated with more active participation as a mentor in own organisation, and leadership of projects or a department. Further, several background factors relating to the organisation had a positive and statistically significant association with APNs' EBHC competence profile or level; (1) teamwork supports EBHC, (2) clear tasks and roles between different experts and (3) evidence is integrated into information systems to support decision‐making (Table 4; Table S2).

### 5.4 Association Between Background Factors and EBHC Competence

We performed four multivariate logistic regression models for both countries (model 1 = personal factors; model 2 = factors related to participation; model 3 = factors related to work tasks; and model 4 = organisational factors) to identify independent association between different background factors and EBHC competence (Table 5).

**TABLE 5:** Multivariate logistic regression models (1–4) of factors associated with participants' EBHC competence in Finland and Singapore.

	Finland^a^	*p*	Singapore^b^	*p*
	Adjusted OR (95% Cl)		Adjusted OR (95% Cl)	
Model 1: Personal factors (*n* = 157)				
The year of completion of the highest degree	**1.07 (1.02–1.13)**	**0.003**	1.06 (0.97–1.15)	0.214
Education level				
Vocational degree (Applied Sciences and University Bachelor's degree)	Reference		Reference	
Master's degree and Doctoral degree (Applied Sciences and University Master's degree)	**4.46 (1.62–12.29)**	**0.004**	0.68 (0.26–1.83)	0.449
Job title	^c^	^c^	^c^	^c^
Current APNs work field				
Primary social‐ and healthcare (Community hospital, Polyclinic and Home Care)	Reference		Reference	
Specialised social‐ and healthcare (Acute care in hospital, Outpatient in hospital)	0.61 (0.24–1.54)	0.294	0.76 (0.19–3.06)	0.701
Model 2: Participation‐related factors, (*n* = 157), reference = did not participate				
Participated in development projects	2.95 (0.93–9.43)	0.067	1.19 (0.37–3.82)	0.989
Participated in research projects	**2.82 (1.04–7.66)**	**0.042**	1.01 (0.19–5.38)	0.765
National professional events (e.g., Events organised by MOH National Nursing Academy)	0.61 (0.23–1.63)	0.321	3.23 (0.76–13.77)	0.113
Scientific conferences	1.68 (0.50–5.62)	0.403	0.44 (0.14–1.34)	0.148
Networks within your own organisation	0.56 (0.21–1.52)	0.255	2.60 (0.86–7.86)	0.091
National network of experts	**5.15 (1.78–14.86)**	**0.002**	1.19 (0.15–9.58	0.870
An international network of experts	0.82 (0.18–3.80)	0.802	1.98 (0.19–20.73)	0.567
Continuing education provided by your own organisation	0.56 (0.23–1.40)	0.216	1.27 (0.35–4.66)	0.712
Continuing education provided by an external organisation	0.64 (0.25–1.61)	0.341	0.75 (0.23–2.43)	0.631
Professional social media (e.g., LinkedIn, Twitter, Facebook)	1.99 (0.74–5.38)	0.172	1.47 (0.29–7.56)	0.645
Model 3: Working‐related factors (*n* = 157), reference = did not working				
In a nursing guideline group	**8.54 (1.96–37.23)**	**0.004**	1.19 (0.34–4.12)	0.787
As an author or contributor of written educational material (e.g., writing a textbook)	1.18 (0.30–4.67)	0.812	3.58 (0.37–34.80)	0.272
As an author or contributor of e‐learning materials (e.g., digital care pathway, e‐learning)	3.08 (0.95–9.98)	0.061	1.64 (0.49–5.47)	0.423
As an educator outside your own organisation (e.g., continuing education, adjunct lecturing, adjunct teaching)	0.68 (0.23–2.06)	0.497	0.55 (0.12–2.51)	0.441
As an educator within your own organisation (e.g., lecturing, simulation training, teaching, bedside clinical teaching)	0.48 (0.15–1.51)	0.211	1.60 (0.51–5.05)	0.420
As a mentor within the organisation (e.g., for research projects, quality improvement projects, new colleagues)	1.06 (4.2–2.67)	0.903	1.22 (0.42–3.52)	0.717
As a consultant in your own organisation	2.15 (0.78–5.88)	0.137	1.12 (0.10–13.27)	0.926
In a leadership role (e.g., project leader, head of department)	**3.95 (1.29–12.12)**	**0.016**	2.68 (0.99–7.22)	0.052
Model 4: Organisational factors (*n* = 157), reference = (connected in the organisation) did not have or did not know if there was				
Evidence‐based healthcare is documented as one of the organisation's strategic plans	1.81 (0.51–6.44)	0.363	3.06 (0.24–39.25)	0.390
Leadership supports the development of evidence‐based healthcare	0.94 (0.25–3.61)	0.930	0.03 (0.00–1.21)	0.062
The organisational culture is positive towards change	0.57 (0.18–1.78)	0.330	14.98 (0.93–241.95)	0.057
Teamwork supports the development of evidence‐based practice	0.70 (0.23–2.14)	0.530	^c^	^c^
I have sufficient resources to develop evidence‐based standard operating practices (SOPs)	**3.67 (1.38–9.73)**	**0.009**	1.89 (0.51–7.04)	0.342
I have an opportunity to be involved in the development of evidence‐based practice	2.69 (0.87–8.29)	0.086	2.22 (0.43–11.55)	0.345
Evidence (e.g., clinical practice guidelines) is readily available in everyday work	1.61 (0.52–5.01)	0.410	0.76 (0.10–5.98)	0.807
My organisation organises Journal clubs	**6.18 (1.71–22.31)**	**0.005**	0.32 (0.06–1.62)	0.166
My organisation has a Research Council	0.42 (0.12–1.48)	0.174	1.88 (0.42–8.43)	0.412
My organisation has clear tasks and roles for different nursing professionals (e.g., registered nurse, enrolled nurse, APN)	1.17 (0.37–3.72)	0.792	0.36 (0.03–3.70)	0.387
My organisation systematically uses existing data (e.g., statistics, registers) for the development of services	0.90 (0.26–3.11)	0.872	0.23 (0.02–2.57)	0.232
The roles of various experts and responsibilities of management in the development of evidence‐based practice are clearly defined in my organisation	1.62 (0.58–4.52)	0.354	**0.08 (0.08–0.72)**	**0.024**
In my organisation, evidence (e.g., clinical practice guidelines) is integrated into information systems to support decision‐making	2.09 (0.82–5.34)	0.122	13.72 (0.92–205.13)	0.058

^a^ Learner *n* = 120 and expert *n* = 37.

^b^ Learner *n* = 66 and expert *n* = 27.

^c^ Analysis did not possible, because all expert responded ‘yes’.

In model 1: Among APNs working in Finland, the EBHC competence was independently related to a higher level of education (*p* = 0.004) and a more recent graduation year of the most recent degree (*p* = 0.003). In model 2: Active participation in research projects (*p* = 0.042) and the national expert network (*p* = 0.002) was independently related to the EBHC competence of APNs operating in Finland. In model 3: Working in a nursing guideline group (*p* = 0.004) and as a project or department leader (*p* = 0.016) was independently related to the EBHC competence of APNs working in Finland. In model 4: The EBHC competence of APNs working in Finland was independently associated with the fact that there were sufficient resources to develop evidence‐based standard operating practices (*p* = 0.009) and that the organisation organised Journal Clubs (*p* = 0.005). Instead, the EBHC competence of those working in Singapore was associated with the fact that the roles of different experts and management responsibilities in the development of evidence‐based practice are clearly defined in my organisation (*p* = 0.024). No statistically significant independent association was found with the EBHC competence of APNs working in Singapore in multivariable logistic regression models 1, 2 and 3.

### 5.5 Differences in Participants' EBHC Competence Between Finland and Singapore

Statistically significant differences were found between APNs working in Finland and Singapore in their self‐assessments of overall EBHC competence (*p* < 0.001) and competence areas of ‘evidence synthesis and transfer’ (*p* < 0.001), ‘evidence implementation’ (*p* = 0.034), and ‘the role of APNs in EBHC’ (*p* < 0.001). APNs working in Singapore assessed their EBHC competence more highly than working in Finland. However, no statistically significant difference was found between APNs in Finland and Singapore in the results of the knowledge test about EBHC, although, the results from APNs in Singapore were better and more consistent than those of APNs in Finland (Table 2).

APNs in Finland had a lower level of education and participated less frequently in development and research projects, scientific conferences, continuing education in own organisation, and worked less frequently in nursing guideline groups, and management tasks than APNs in Singapore. There were statistically significant differences between Finnish and Singaporean organisational factors that may associated with APNs' EBHC competence. For example, APNs working in Singapore estimate more often than APNs in Finland that in their organisation EBHC is documented in the organisation's strategy; leadership supports EBHC's development; the organisational culture is positive towards change; teamwork supports evidence‐based practice (EBP); they have the opportunity to participate in the development of EBP; the organisation has a Journal clubs; the organisation has a research council; different nursing professionals have clear tasks and roles; the roles of different experts and the management's responsibilities in the development of EBP are clearly defined; and the evidence is integrated into information systems (Table 1; Table S3).

## 6 Discussion

This study aimed to describe the EBHC competence of APNs, and the factors associated with it in Finland and Singapore. We found significant variation in APNs' levels of EBHC competence across sub‐dimensions and competence profiles, especially in Finland. This suggests that the EBHC competence of APNs needs further development in Finland and Singapore. Among the APNs of both countries, EBHC competence was strongest in ‘the knowledge needs related to global health’ sub‐dimension, which includes identifying the need for information and critical examination of practice and commitment to implementing EBHC. These results were consistent with the findings of the systematic review (Ylimäki et al. 2024). The weakest EBHC competence area among APNs was ‘synthesis and transfer evidence’, which partially corresponds to previous findings (Ylimäki et al. 2024). On the other hand, in this study, APNs did not find generating evidence generation as challenging as identified in a previously conducted systematic review (Ylimäki et al. 2024).

The EBHC competence of APNs in Singapore was statistically higher than that of Finnish APNs, apart from the sub‐dimensions ‘the knowledge needs related to global health’ and ‘evidence generation’. Results from the EBHC knowledge test were also higher in Singapore, although the difference was not statistically significant. APNs in Singapore achieved more consistent results on the EBHC knowledge test than those in Finland, in part because Finland does not define a specific educational requirement for nurses performing advanced and/or independent tasks, and the education of APNs in Finland is not as standardised as in Singapore. It should be noted that the country (Finland, Singapore) was not an independently competence‐related variable in the multivariate logistic regression, so it does not explain differences in APNs' EBHC competence between countries.

This study found that the EBHC competence profiles of Finnish APNs were associated with several factors including education level; year of highest education; job title; current place of practice; participation in development and research projects (Table 3), scientific conferences, in own organisation network and a national expert network; following professional social media; working in a nursing guideline group; producing or contributing to educational material; and being mentor, consultant and leadership (Table S1). In addition, several organisational factors were associated with the Finnish APNs' EBHC competence profiles (see Table S1). The EBHC competence profiles of APNs working in Singapore were associated with gender, highest education year (Table 3), working as a mentor and leadership (Table S2). In addition, the EBHC competence profiles of APNs working in Singapore were associated with teamwork supporting evidence‐based nursing, clear roles and responsibilities of different experts in developing EBHC and integrating evidence into information systems (Table S2).

It is important to note that the differences in background factors between APNs in Finland and Singapore may partly explain the observed disparities between the countries. The competence differences between the countries could partially be explained by (1) the education level in this study and also internationally (Legatum Institute 2024; see Tables 3, 4 and 5), (2) the emphasis on the field of specialised healthcare in the Singaporean research material (See Tables 3 and 4), (3) more active participation in research and development projects (See Tables 3, 4 and 5) and (4) activities supporting the implementation of the healthcare system EBHC in this study and also internationally (International Citizens Insurance 2024; see Tables S1, S2 and 5).

A higher educational level of APNs working in Finland was independently associated with higher EBHC competence. This observation is along with findings from previous studies (Immonen et al. 2022; Ylimäki et al. 2024). However, the difference in education level was not statistically associated with the EBHC competence of APNs working in Singapore. Therefore, this finding confirms the previous understanding (Holloway et al. 2018) that it is important to identify what the competence level is like, and which factors are associated with the competence in each context before developing the competence.

Another key factor associated with the EBHC competence of APNs operating in Finland and Singapore was related to the year in which the participants had graduated their highest degree. Those who graduated longer ago registered the lowest level of EBHC competence. The JBI Model of EBHC was created in 2005 (Jordan et al. 2019), meaning it is relatively new, and its integration into nursing education has taken some time. This may partly explain why the EBHC competence level of recent graduates is better. The same result was also identified in the study by Immonen et al. (2024), whose target population was Finnish social and healthcare educators. In addition, in both countries the task descriptions of APNs are quite new, and the educating of APNs is developing, which may be partly related to the better EBHC competence of recent graduates.

The level of EBHC competence of Finnish APNs was strengthened by participation in research projects, the same result was also observed among Finnish social‐ and healthcare educators (Immonen et al. 2024). Woo et al. (2019) in their study found that the time APNs spend in their work on a certain sub‐dimension is positively correlated with the competence of that sub‐dimension. This study found that participation in research projects and development projects was very common among APNs working in Singapore, while only less than fifth of APNs working in Finland participated in research projects, which confirms Woo et al. (2019) observation. In addition, participating in national network of experts, working in a nursing guideline group and leading projects or personnel strengthened Finnish APNs' EBHC competence. Other previous studies have not examined the relationship between evidence generation, transfer, nursing leadership and the level of EBHC competence.

These new findings are important in the development of EBHC competence, as EBHC competence includes a lot of competence needed in research and development. These roles are essential to APN's mission and contribute to the competencies associated with their primary role. APNs have a unique role in the health sector in promoting and developing evidence‐based healthcare within their organisation (Schober et al. 2020). This includes developing the EBHC competencies of other professional groups through education, mentoring and consultation. They are also responsible for monitoring and evaluating the implementation of evidence‐based practices in clinical settings (Jokiniemi et al. 2020; Schober et al. 2020; Ylimäki et al. 2022; Ylimäki et al. 2024). To fulfil this role, APNs need to have a deep understanding of the JBI Model of EBHC and be competent in every step of the EBHC process because without care according to the EBHC model, the implementation of EBN is not possible.

### 6.1 Strengths and Limitations

This study's strength is that those results from the various instruments support each other. EBHC competence was measured using a self‐assessment instrument (scale 1–5) and the EBHC knowledge test. The results from the EBHC competence self‐assessment and the EBHC knowledge test were consistent for each profile. For example, Profile A evaluated the EBHC competence as the highest and had the best results in EBHC knowledge test. This shows that the results of the instruments are in line with each other. A further strength was that the EBHC‐Comp‐APN instrument's reliability according to Cronbach's alpha proved excellent in both the Finnish and English instruments (Finnish α = 0.987, English α = 0.982) and the instrument's sub‐dimensions (Finnish α = 0.937–0.971, English α = 0.912–0.964). In addition, a statistician was involved in the analysis.

The generalizability of the study is limited by the small number of registered APNs in both Finland and Singapore. Both countries have well‐developed and high‐quality healthcare services, and the roles of APNs are somewhat established. This study's target population included all nursing professionals who provide advanced and independent care because this study described the current level of EBHC competence of all advanced and independent nursing professionals. This may have increased the observed variability in EBHC competence and educational background.

The complete response rate for the entire target population could not be calculated due to insufficient registered data on the number of registered and senior nurses with advanced job descriptions. The relatively low response rate in Singapore may be explained by several studies targeting the same target population over the same period. In Finland, some of the data collection took place during the summer vacation. Nine unfinished questionnaires were excluded. The length of the survey may explain why these were unfinished. In evaluating the generalizability, and usability of the research results, the participants' characteristics and the well‐developed and high‐quality healthcare services of the particular countries studied must be taken into consideration.

The smaller size of the data from Singapore may contribute to the fact that not all factors related to APNs' EBHC competence were identified using cluster analysis and logistic regression models based on dichotomous division. Due to the limitations mentioned above, it is important to continue research in the field.

### 6.2 Recommendations for Further Research

It would also be valuable for future research to consider confounding factors which affect EBHC competence. In the future, it is important to continue researching APNs' EBHC competence profiles and related factors, considering all key areas of EBHC, as this was the first study to map this. In addition, there is a need for systematic research on how the previous experience of generating, synthesis, transferring and implementing evidence and leading the implementation, is associated with the EBHC competence of APNs.

### 6.3 Implications for Policy and Practice

Healthcare organisations should consider the EBHC competence needs identified in this study and the factors associated with EBHC competence in the development of APNs' EBHC competence. In APNs' education should comprehensively consider the EBHC model and integrate it into the teaching content. In addition, there are indications that APNs could benefit from practical training during education, e.g., generating research, searching for evidence, evidence synthesis, evidence transfer and implementation. Practicing the competence needed in future work should be integrated in the teaching. Nationally, APNs' tasks descriptions and job titles should be defined and validated to harmonise and clarify APNs' duties and the competence expected of them.

## 7 Conclusion

The level of EBHC competence of APNs varied from poor to good across profiles. The weakest competence was in ‘evidence synthesis and transfer’. Special attention should be paid to this so that APNs can promote the use of reliable evidence such as systematic reviews, national nursing guidelines and other evidence summaries among healthcare providers. Personal and organisational factors were associated with APNs' EBHC competence. Healthcare organisations should therefore provide APNs with sufficient resources and opportunities to participate in relevant practices and integrate evidence into information systems to build their EBHC competence. APNs should also be encouraged to participate in research projects and a national expert network, work in a nursing guideline group and take on leadership‐related roles (leading a development or research project or department) to develop their EBHC competencies. In addition, at the national level, APNs, education and healthcare organisations, and policymakers should agree on a sufficient level of education to manage a wide‐ranging and independent role and to promote EBHC at the organisational level. The results of this study can be used to develop continuing and basic education on EBHC for APNs or profile groups of APNs and increase organisation knowledge of how to support EBHC competence among APNs.

## Author Contributions

Made substantial contributions to conception and design, or acquisition of data or analysis and interpretation of data: Saija Ylimäki, Anne Oikarinen, Anna‐Maria Tuomikoski, Brigitte Woo, Kristina Mikkonen, Wentao Zhou, Maria Kääriäinen; Involved in drafting the article or revising it critically for important intellectual content: Saija Ylimäki, Anne Oikarinen, Anna‐Maria Tuomikoski, Brigitte Woo, Wentao Zhou, Heidi Parisod, Sami Sneck, Hannu Vähänikkilä, Maria Kääriäinen; Given final approval of the version to be published. Each author should have participated sufficiently in the work to take public responsibility for appropriate portions of the content: Saija Ylimäki, Anne Oikarinen, Anna‐Maria Tuomikoski, Brigitte Woo, Hannu Vähänikkilä, Kristina Mikkonen, Wentao Zhou, Heidi Parisod, Sami Sneck, Maria Kääriäinen; Agreed to be accountable for all aspects of the work in ensuring that questions related to the accuracy or integrity of any part of the work are appropriately investigated and resolved: Saija Ylimäki, Anne Oikarinen, Anna‐Maria Tuomikoski, Brigitte Woo, Hannu Vähänikkilä, Kristina Mikkonen, Wentao Zhou, Heidi Parisod, Sami Sneck, Maria Kääriäinen.

## Conflicts of Interest

The authors declare no conflicts of interest.

## Peer Review

The peer review history for this article is available at https://www.webofscience.com/api/gateway/wos/peer‐review/10.1111/jan.16771.

## Correctness of Statistics Analysis

There is a statistician on the author team and state which author.

## Research Permits

The National University of Singapore's Institutional Review Board (NUS‐IRB‐2023‐645) permission from Singapore. In Finland, the ethical review was not required, but organisation‐specific research permits were required and granted by the organisations.

## Publicly Available Research Data

In addition to the tables in the cross‐sectional study, two additional tables are publicly available. These are Supporting Information.

## Supporting information


Data S1.

Data S2.

## Data Availability

The data that supports the findings of this study are available in the Supporting Information of this article.
