# Supplementary material for: Advanced Practice Nurses' Evidence‐Based Healthcare Competence and Associated Factors in Finland and Singapore—A Cross‐Sectional Study
Source: J Adv Nurs. 2025 Jan 24;81(10):6417–31. doi: 10.1111/jan.16771 (PMC12460981; doi:10.1111/jan.16771)
Supplement: Supplementary file 2 — Data S2. [file JAN-81-6417-s002.docx]

Table S1. The connection of participation, working, and organizational factors to the EBHC competence profiles of the participants (n=157) in Finland

|  | ***Profile A*** (n=50) | ***Profile B*** (n=58) | Profile C (n=49) | ***Total*** | p |
| --- | --- | --- | --- | --- | --- |
| **Participated in last two years, n (%)** |  |  |  |  |  |
| National professional events (e.g. Events organized by MOH National Nursing Academy) | 23 (46.0) | 27 (46.6) | 16 (32.7) | 66 (42.0) | 0.275* |
| Scientific conferences | 15 (30.0) | 6 (10.3) | 0 (0.0) | 21 (13.4) | **<0.001*** |
| Networks within your own organisation | 29 (58.0) | 35 (60.3) | 17 (34.7) | 81 (51.6) | **0.017*** |
| National network of experts | 24 (48.0) | 12 (20.7) | 6 (12.2) | 42 (26.8) | **<0.001*** |
| An international network of experts | 6 (12.0) | 4 (6.9) | 2 (4.1) | 12 (7.6) | 0.360^ |
| Continuing education provided by your own organisation. | 28 (56.0) | 34 (58.6) | 29 (59.2) | 91 (58.0) | 0.958* |
| Continuing education provided by an external organisation. | 33 (66.0) | 31 (53.4) | 31 (63.3) | 95 (60.5) | 0.365* |
| Professional social media (e.g. LinkedIn, Twitter, Facebook) | 35 (70.0) | 24 (41.4) | 18 (36.7) | 77 (49.0) | **0.001*** |
| Further academic education at university (towards a doctorate) | 2 (4.0) | 0 (0.0) | 0 (0.0) | 2 (1.3) | 0.196^ |
| **I have worked, n (%)** |  |  |  |  |  |
| In a nursing guideline group | 10 (20.0) | 2 (3.4) | 1 (2.0) | 13 (8.3) | **0.002^** |
| As an author or contributor of written educational material (e.g. writing a textbook) | 11 (22.0) | 3 (5.2) | 2 (4.1) | 16 (10.2) | **0.004*** |
| As an author or contributor of e-learning materials (e.g. digital care pathway, e-learning) | 18 (36.0) | 12 (20.7) | 0 (0.0) | 30 (19.1) | **<0.001*** |
| As an educator outside your own organisation (e.g. continuing education, adjunct lecturing, adjunct teaching) | 15 (30.0) | 16 (27.6) | 10 (20.4) | 41 (26.1) | 0.552* |
| As an educator within your own organisation (e.g. lecturing, simulation training, teaching, bedside clinical teaching) | 36 (72.0) | 34 (58.6) | 24 (49.0) | 94 (59.9) | 0.064* |
| As a mentor within the organisation (e.g. for research projects, quality improvement projects, new colleagues) | 19 (38.0) | 26 (44.8) | 9 (18.4) | 54 (34.4) | **0.013*** |
| As a consultant in your own organisation | 21 (42.0) | 16 (27.6) | 8 (16.3) | 45 (28.7) | **0.018*** |
| In a leadership role (e.g. project leader, head of department) | 16 (32.0) | 4 (6.9) | 5 (10.2) | 25 (15.9) | **<0.001*** |
| **I know that the following items come true in my organisation, n (%)** |  |  |  |  |  |
| Evidence-based healthcare is documented as one of the organisation’s strategic plans. | 42 (84.0) | 45 (77.6) | 25 (51.0) | 112 (71.3) | **<0.001*** |
| Leadership supports the development of evidence-based healthcare. | 39 (78.0) | 43 (74.1) | 26 (53.1) | 108 (68.8) | **0.014*** |
| The organisational culture is positive towards change. | 35 (70.0) | 44 (75.9) | 31 (63.3) | 110 (70.1) | 0.359* |
| Teamwork supports the development of evidence-based practice. | 36 (72.0) | 48 (82.8) | 30 (61.2) | 114 (72.6) | **0.049*** |
| I have sufficient resources to develop evidence-based standard operating practices (SOPs). | 32 (64.0) | 23 (39.7) | 16 (32.7) | 71 (45.2) | **0.004*** |
| I have an opportunity to be involved in the development of evidence-based practice. | 41 (82.0) | 35 (60.3) | 19 (38.8) | 95 (60.5) | **<0.001*** |
| Evidence (e.g. clinical practice guidelines) is readily available in everyday work. | 38 (76.0) | 40 (69.0) | 33 (67.3) | 111 (70.7) | 0.636* |
| My organisation organizes Journal clubs. | 24 (48.0) | 17 (29.3) | 6 (12.2) | 47 (29.9) | **<0.001*** |
| My organisation has a Research Council. | 19 (38.0) | 24 (41.4) | 9 (18.4) | 52 (33.1) | **0.030*** |
| My organisation has clear tasks and roles for different nursing professionals (e.g. registered nurse, enrolled nurse, APN) | 10 (20.0) | 16 (27.6) | 5 (10.2) | 31 (19.7) | 0.085* |
| My organisation systematically uses existing data (e.g. statistics, registers) for the development of services. | 36 (72.0) | 39 (67.2) | 24 (49.0) | 99 (63.1) | **0.044*** |
| The roles of various experts and responsibilities of management in the development of evidence-based practice are clearly defined in my organization. | 35 (70.0) | 41 (70.7) | 41 (83.7) | 117 (74.5) | 0.224* |
| In my organisation, evidence (e.g. clinical practice guidelines) is integrated into information systems to support decision-making. | 23 (46.0) | 21 (36.2) | 16 (32.7) | 60 (38.2) | 0.369* |

Pearson Chi-Square Test, *Pearson Chi-Square Test, ^Fisher-Freeman-Halton Exact Test

Table S2. The connection of participation, working, and organizational factors to the EBHC competence profiles of the participants (n=93) in Singapore

|  | Profile A (n=27) | Profile B (n=44) | Profile C (n=22) | ***Total*** | ***p*** |
| --- | --- | --- | --- | --- | --- |
| **Participated in last two years, n (%)** |  |  |  |  |  |
| National professional events (e.g. Events organized by MOH National Nursing Academy) | 6 (22.2) | 6 (13.6) | 0 (0.0) | 12 (12.9) | 0.058^ |
| Scientific conferences | 14 (51.9) | 24 (54.5) | 10 (45.5) | 48 (51.6) | 0.830* |
| Networks within your own organisation | 13 (48.1) | 20 (45.5) | 5 (22.7) | 38 (40.9) | 0.155* |
| National network of experts | 4 (14.8) | 5 (11.4) | 0 (0.0) | 9 (9.7) | 0.162^ |
| An international network of experts | 3 (11.1) | 3 (6.8) | 0 (0.0) | 6 (6.5) | 0.357^ |
| Continuing education provided by your own organisation. | 22 (81.5) | 35 (79.5) | 15 (68.2) | 72 (77.4) | 0.507* |
| Continuing education provided by an external organisation. | 10 (37.0) | 19 (43.2) | 10 (45.5) | 39 (41.9) | 0.828* |
| Professional social media (e.g. LinkedIn, Twitter, Facebook) | 3 (11.1) | 4 (9.1) | 3 (13.6) | 10 (10.8) | 0.909^ |
| Further academic education at university (towards a doctorate) | 1 (3.7) | 1 (2.3) | 2 (9.1) | 4 (4.3) | 0.355^ |
| **I have worked, n (%)** |  |  |  |  |  |
| In a nursing guideline group | 8 (29.6) | 9 (20.5) | 2 (9.1) | 19 (20.4) | 0.198* |
| As an author or contributor of written educational material (e.g. writing a textbook) | 3 (11.1) | 2 (4.5) | 0 (0.0) | 5 (5.4) | 0.195^ |
| As an author or contributor of e-learning materials (e.g. digital care pathway, e-learning) | 8 (29.6) | 5 (11.4) | 4 (18.2) | 17 (18.3) | 0.153^ |
| As an educator outside your own organisation (e.g. continuing education, adjunct lecturing, adjunct teaching) | 4 (14.8) | 6 (13.6) | 5 (22.7) | 15 (16.1) | 0.670^ |
| As an educator within your own organisation (e.g. lecturing, simulation training, teaching, bedside clinical teaching) | 19 (70.4) | 27 (61.4) | 14 (63.6) | 60 (64.5) | 0.725* |
| As a mentor within the organisation (e.g. for research projects, quality improvement projects, new colleagues) | 12 (44.4) | 18 (40.9) | 3 (13.6) | 33 (35.5) | **0.049*** |
| As a consultant in your own organisation | 2 (7.4) | 2 (4.5) | 0 (0.0) | 4 (4.3) | 0.569^ |
| In a leadership role (e.g. project leader, head of department) | 16 (59.3) | 14 (31.8) | 5 (22.7) | 35 (37.6) | **0.019*** |
| **I know that the following items come true in my organisation, n (%)** |  |  |  |  |  |
| Evidence-based healthcare is documented as one of the organisation’s strategic plans. | 25 (92.6) | 39 (88.6) | 20 (90.9) | 84 (90.3) | 0.905^ |
| Leadership supports the development of evidence-based healthcare. | 26 (96.3) | 43 (97.7) | 19 (86.4) | 88 (94.6) | 0.142^ |
| The organisational culture is positive towards change. | 26 (96.3) | 34 (77.3) | 18 (81.8) | 78 (83.9) | 0.085^ |
| Teamwork supports the development of evidence-based practice. | 27 (100.0) | 37 (84.1) | 18 (81.8) | 82 (88.2) | **0.050^** |
| I have sufficient resources to develop evidence-based standard operating practices (SOPs). | 19 (70.4) | 23 (52.3) | 9 (40.9) | 51 (54.8) | 0.107* |
| I have an opportunity to be involved in the development of evidence-based practice. | 23 (85.2) | 33 (75.0) | 14 (63.6) | 70 (75.3) | 0.230* |
| Evidence (e.g. clinical practice guidelines) is readily available in everyday work. | 24 (88.9) | 34 (77.3) | 17 (77.3) | 75 (80.6) | 0.491* |
| My organisation organizes Journal clubs. | 20 (74.1) | 35 (79.5) | 19 (86.4) | 74 (79.6) | 0.639* |
| My organisation has a Research Council. | 21 (77.8) | 32 (72.7) | 18 (81.8) | 71 (76.3) | 0.739* |
| My organisation has clear tasks and roles for different nursing professionals (e.g. registered nurse, enrolled nurse, APN) | 25 (92.6) | 38 (86.4) | 17 (77.3) | 80 (86.0) | 0.320^ |
| My organisation systematically uses existing data (e.g. statistics, registers) for the development of services. | 22 (81.5) | 32 (72.7) | 13 (59.1) | 67 (72.0) | 0.250* |
| The roles of various experts and responsibilities of management in the development of evidence-based practice are clearly defined in my organization. | 21 (77.8) | 26 (59.1) | 6 (27.3) | 40 (43.0) | **0.001*** |
| In my organisation, evidence (e.g. clinical practice guidelines) is integrated into information systems to support decision-making. | 25 (92.6) | 32 (72.7) | 11 (50.0) | 68 (73.1) | **0.004*** |

Pearson Chi-Square Test, *Pearson Chi-Square Test, ^Fisher-Freeman-Halton Exact Test

Table S3. The connection of participation, working, and organizational factors to the EBHC competence profiles of the participants in Finland and Singapore

|  | ***Finland*** (n=157) | Singapore (n=99) | ***p*** |
| --- | --- | --- | --- |
| **Participated in last two years, n (%)** |  |  |  |
| National professional events (e.g. Events organized by MOH National Nursing Academy) | 66 (42.0) | 13 (13.1) | **<0.001*** |
| Scientific conferences | 21 (13.4) | 49 (49.5) | **<0.001*** |
| Networks within your own organisation | 81 (51.6) | 39 (39.4) | **0.072*** |
| National network of experts | 42 (26.8) | 9 (9.1) | **<0.001*** |
| An international network of experts | 12 (7.6) | 6 (6.1) | 0.803* |
| Continuing education provided by your own organisation. | 91 (58.0) | 77 (77.8) | **<0.001*** |
| Continuing education provided by an external organisation. | 95 (60.5) | 42 (42.4) | **0.007*** |
| Professional social media (e.g. LinkedIn, Twitter, Facebook) | 77 (49.0) | 10 (10.1) | **0.001*** |
| Further academic education at university (towards a doctorate) | 2 (1.3) | 4 (4.0) | 0.210^ |
| **I have worked, n (%)** |  |  |  |
| In a nursing guideline group | 13 (8.3) | 19 (19.2) | **0.012*** |
| As an author or contributor of written educational material (e.g. writing a textbook) | 16 (10.2) | 5 (5.1) | 0.167* |
| As an author or contributor of e-learning materials (e.g. digital care pathway, e-learning) | 30 (19.1) | 17 (17.2) | 0.743* |
| As an educator outside your own organisation (e.g. continuing education, adjunct lecturing, adjunct teaching) | 41 (26.1) | 15 (15.2) | 0.044* |
| As an educator within your own organisation (e.g. lecturing, simulation training, teaching, bedside clinical teaching) | 94 (59.9) | 63 (63.6) | 0.599* |
| As a mentor within the organisation (e.g. for research projects, quality improvement projects, new colleagues) | 54 (34.4) | 36 (36.4) | 0.789* |
| As a consultant in your own organisation | 45 (28.7) | 5 (5.1) | **<0.001*** |
| In a leadership role (e.g. project leader, head of department) | 25 (15.9) | 38 (38.4) | **<0.001*** |
| **I know that the following items come true in my organisation, n (%)** |  |  |  |
| Evidence-based healthcare is documented as one of the organisation’s strategic plans. | 112 (71.3) | 90 (90.9) | **<0.001*** |
| Leadership supports the development of evidence-based healthcare. | 108 (68.8) | 93 (93.9) | **<0.001*** |
| The organisational culture is positive towards change. | 110 (70.1) | 82 (82.8) | **0.026*** |
| Teamwork supports the development of evidence-based practice. | 114 (72.6) | 87 (87.9) | **0.005*** |
| I have sufficient resources to develop evidence-based standard operating practices (SOPs). | 71 (45.2) | 54 (54.5) | 0.159* |
| I have an opportunity to be involved in the development of evidence-based practice. | 95 (60.5) | 75 (75.8) | **0.014*** |
| Evidence (e.g. clinical practice guidelines) is readily available in everyday work. | 111 (70.7) | 78 (78.8) | 0.189* |
| My organisation organizes Journal clubs. | 47 (29.9) | 78 (78.8) | **<0.001*** |
| My organisation has a Research Council. | 52 (33.1) | 74 (74.7) | **<0.001*** |
| My organisation has clear tasks and roles for different nursing professionals (e.g. registered nurse, enrolled nurse, APN) | 31 (19.7) | 84 (84.8) | **<0.001*** |
| My organisation systematically uses existing data (e.g. statistics, registers) for the development of services. | 99 (63.1) | 71 (71.7) | 0.175* |
| The roles of various experts and responsibilities of management in the development of evidence-based practice are clearly defined in my organization. | 40 (25.5) | 57 (61.3) | **<0.001*** |
| In my organisation, evidence (e.g. clinical practice guidelines) is integrated into information systems to support decision-making. | 60 (38.2) | 72 (72.7) | **<0.001*** |

Pearson Chi-Square Test, *Pearson Chi-Square Test, ^Fisher-Freeman-Halton Exact Test
